# P-1466. Prevalence of Antimicrobial Resistance among Invasive *Escherichia coli* Disease Patients in the United States

**DOI:** 10.1093/ofid/ofae631.1638

**Published:** 2025-01-29

**Authors:** Sanjukta Basu, Ziyan (Jenny) Wei, Atara Laor, Liga Bennetts, Antoine El Khoury, Nina Ahmad, Jeroen Geurtsen, Maureen P Neary

**Affiliations:** Amaris Consulting, Toronto, Ontario, Canada; Amaris Consulting, Toronto, Ontario, Canada; Amaris Consulting, Toronto, Ontario, Canada; Amaris Consulting, Toronto, Ontario, Canada; Janssen Global Services LLC, Titusville, New Jersey; Janssen Global Services LLC, Titusville, New Jersey; Janssen Vaccines & Prevention BV, Leiden, Netherlands; Janssen Global Services, LLC, Raritan, New Jersey

## Abstract

**Background:**

Invasive *E. coli* disease (IED) poses a substantial public health concern globally. Anti-microbial resistance (AMR) displayed by some *E. coli* strains further limits the treatment options. We sought to identify and synthesize evidence on prevalence of AMR among adult IED patients in the United States.

Figure 1.

**Figure:**
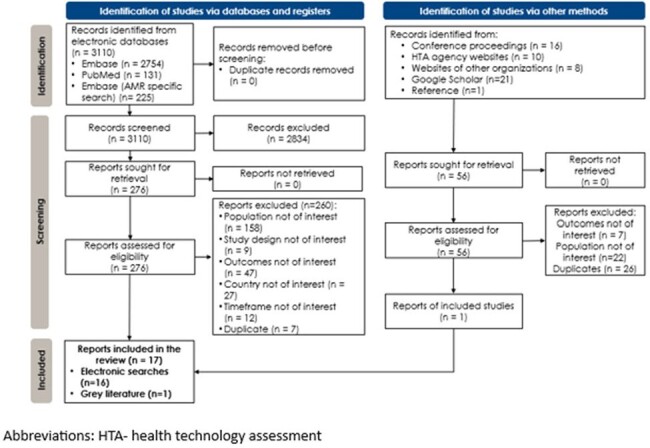

PRISMA flowchart

**Methods:**

A systematic literature review was conducted in EMBASE, MEDLINE, and MEDLINE In-Process databases to identify studies of prevalence of AMR *E. coli* among adult IED patients, published January. 2010–September. 2023.

Figure 2.

**Figure:**
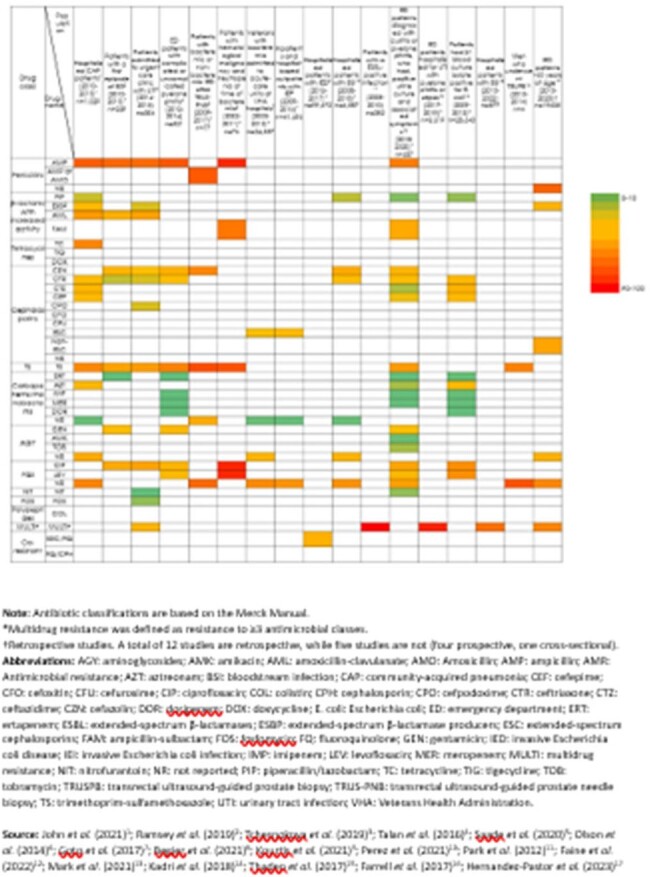

Antimicrobial resistance of E. coli isolates to antibiotics across patient groups

**Results:**

Seventeen publications met eligibility criteria and were included (**Fig. 1**). The review reports study-specific prevalence from these included studies. Variation in AMR was observed across study designs (retrospective: 12; cross-sectional: 1; prospective: 4) with varying population characteristics, and 19-year time period (2002–2020) (**Fig. 2**). Four studies had high prevalence (≥50%) of ampicillin-resistant *E. coli*. Extended-spectrum cephalosporin resistance (ESC-R) increased over time in two studies (2003–2007: 4.1% & 2008–2013: 7.9% among veterans with bacteremia; and 2009: 5.4% & 2016: 12.9% for IED-related visits). One study reported higher prevalence of *E. coli* AMR among older as opposed to younger patients for ESC-R (18-49 years: 6%; > 65 years: 10%) and FQ-R (18-49 years: 18%; > 65 years: 31%). Similar trends were observed for *E. coli* multidrug resistance (≤69 years: 10% vs > 69 years: 19%) in one study. FQ/ESC co-resistance increased over time, from 11% in 2013 to 14% in 2017 (p < .0001). In one study, AMR rates were lower during Summer for amoxicillin/clavulanic acid (17% vs 29%; p=.02), cefazolin (6% vs 19%; p < .001), trimethoprim/sulfamethoxazole (9% vs 27%; p < .001) and ceftriaxone (2% vs 6%; p=.04). Prevalence of ampicillin resistance was not seasonal. Regional variation in AMR was observed, with FQ-R varying between 10.5% in Denver Health Clinic to 28.9% in Cleveland Clinic. Prevalence of FQ/ESC co-resistance varied geographically; highest prevalence ( > 13%) occurred in several regions.

**Conclusion:**

Prevalence of AMR *E. coli* in the US increased overall, in certain high-risk groups, over time, with also regional and seasonal variation. There is an unmet need for additional data on AMR prevalence in IED, and a critical need for preventive measures.

**Disclosures:**

**Sanjukta Basu, Ph.D**, Amaris Consulting: Advisor/Consultant **Ziyan (Jenny) Wei, Msc**, Amaris Consulting: Advisor/Consultant **Atara Laor, BSc.**, Amaris Consulting: Advisor/Consultant **Liga Bennetts, PhD**, Amaris Consulting: Advisor/Consultant **Antoine El Khoury, PhD**, Janssen Global Services: Advisor/Consultant|Janssen Global Services, LLC: Employee of Janssen Global Services, LLC **Nina Ahmad, MD**, Janssen Global Services, LLC: Employee of Janssen Global Services, LLC **Jeroen Geurtsen, PhD**, Janssen Vaccines & Prevention BV: Advisor/Consultant|Janssen Vaccines & Prevention BV: Employee of Janssen Vaccines & Prevention BV **Maureen P. Neary, PhD, MS**, Janssen Global Services, LLC: Employee of Janssen Global Services, LLC

